# Investigation and Application of Risk Factors of Macrosomia Based on 10,396 Chinese Pregnant Women

**DOI:** 10.3389/fendo.2022.837816

**Published:** 2022-04-26

**Authors:** Xiaosong Yuan, Xiaoya Han, Chenbo Jia, Wei Long, Huiyan Wang, Bin Yu, Jun Zhou

**Affiliations:** ^1^ Department of Medical Genetics, Changzhou Maternal and Child Health Care Hospital, Changzhou Medical Center, Nanjing Medical University, Changzhou, China; ^2^ Department of Obstetrics and Gynecology Changzhou Maternal and Child Health Care Hospital, Changzhou Medical Center, Nanjing Medical University, Changzhou, China

**Keywords:** D-dimer, blood lipids, birthweight, macrosomia, large for gestational age, prediction

## Abstract

**Objective:**

The objective of this study was to examine the association of fetal macrosomia with maternal D-dimer and blood lipid levels, and explore whether D-dimer and blood lipids, either alone or in combination with traditional risk factors at hospital birth, could be used to predict subsequent delivery of macrosomia.

**Methods:**

From April 2016 to March 2017, 10,396 women with singleton pregnancy giving birth at around 28–41 weeks of gestation were recruited into the present study. D-dimer and blood lipid levels were measured at hospital admission; and data on birth outcomes were obtained from hospital records.

**Results:**

Multivariate logistic regression analysis showed that D-dimer, triglyceride and HDL-C levels were significantly associated with risk of macrosomia independent of traditional risk factors (for D-dimer: adjusted OR: 1.33, 95% CI, 1.23–1.43; for triglyceride: adjusted OR: 1.14, 95% CI, 1.05–1.23; for HDL-C: adjusted OR: 0.35, 95% CI, 0.24–0.51, all *P <*0.01). More importantly, incorporating D-dimer and blood lipids into the traditional model significantly increased the area under curve (AUC) for prediction of macrosomia (0.783 vs. 0.811; *P <*0.01).

**Conclusion:**

Our study demonstrates that maternal D-dimer, triglyceride, and HDL-C levels before hospital birth could be significant and independent of risk factors of fetal macrosomia. Therefore, combining D-dimer and blood lipid levels with traditional risk factors might improve the ability to predict macrosomia in gestational diabetes mellitus and normal pregnancies.

## Introduction

Macrosomia with birth weight >4,000 g are at high risk of adverse perinatal outcomes, namely, shoulder dystocia, birth injury and asphyxia, and perinatal death (1, 2). Macrosomia offsprings also have an excess risk of future hypertension, type 2 diabetes and obesity (3, 4). Prenatal prediction of macrosomia makes it possible to intervene through caesarean section or labor induction, thereby preventing macrosomia birth or complications of vaginal delivery of macrosomic fetus. In clinical practice, physical examination (maternal abdomen and fundal height) and ultrasound assessment are usually used to estimate fetal macrosomia. However, the best approach for detecting fetal macrosomia is uncertain, since previous studies from the general population demonstrated low predictive values of these methods (5). The latest research area that has the potential to improve the prediction of fetal macrosomia is the field of biomarkers in which a number of maternal and fetal biomarkers have previously demonstrated an association with fetal birthweight or macrosomia in pregnancies (6, 7).

D-dimer, a smaller fragment of fibrinogen/fibrin degradation products, increases gradually during pregnancy (8, 9). D-dimer is one of the most valuable biomarkers to exclude venous thromboembolism disorders in pregnant women and non-pregnant population (10, 11). Previous studies have shown that D-dimer levels might predict a higher risk of pregnancy failure in women who underwent *in-vitro* fertilization and guide anticoagulant treatment in recurrent pregnancy loss associated with antiphospholipid syndrome; the high levels of D-dimer detected at more than 20 weeks of gestation were related to the severity of preeclampsia and maternal serum D-dimer combined with alpha-fetoprotein (AFP) and free β-subunit of human chorionic gonadotropin (free β-hCG) at the second trimester of pregnancy might be used to predict hypertensive disorders of pregnancy (12–15). However, there has been little research on the associations of maternal D-dimer level with fetal birthweight.

At the beginning of the 12th week of gestation, especially in the second and third trimesters, maternal blood lipids, namely, triglycerides (TG), total cholesterol (TC), high-density lipoprotein cholesterol (HDL-C), and low-density lipoprotein cholesterol (LDL-C), increase significantly (16, 17). Previous cohort studies showed that maternal TG and/or HDL-C levels measured either during early pregnancy or late pregnancy are associated with the risk of macrosomia and/or large for gestational age (LGA) birth (18–20). However, to our knowledge, few studies have combined D-dimer, blood lipids, and routine risk factors for prenatal prediction of macrosomia and/or LGA newborns during the hospital admission for delivery. The objective of this study was to evaluate the ability of maternal D-dimer and blood lipid levels before delivery to predict macrosomia and/or LGA birth, and to determine whether combining D-dimer and blood lipids with routine risk factors could improve the predictive performance.

## Methods

### Study Participants

A cohort of 12,627 consecutive pregnant women who were admitted to the Changzhou Maternity and Child Health Care Hospital affiliated to Nanjing Medical University for their delivery of singleton were recruited to our retrospective observational study from April 2016 and March 2017. Ethical approval was obtained from the Ethics Committee of our hospital (No. ZD201803). All eligible participants provided written informed consents. Among the women who were recruited, 2,231 who presented with major pre-gestational disease, namely, diabetes mellitus (type 1 or 2), chronic hypertension, chronic heart, liver and kidney diseases, immune rheumatic disease, thyroid diseases, and syphilis, which might contribute to an increase in D-dimer and blood lipid levels (n = 488) or without D-dimer and blood lipid levels at hospital admission (n = 328), or fetal malformation and stillbirth (n = 72), or abortion (n = 24), or ICP (n = 738), or PE (n = 379), or PIH (n = 202) were excluded from the present study.

### Laboratory Assessment and Data Collection

Maternal blood specimens were collected from the participants at their hospital admission for delivery (median = 39 weeks; minimum: 28 weeks; maximum: 41 weeks). Serum and plasma samples were routinely collected before active labor and assayed for levels of blood lipid levels and D-dimer through latex enhanced immunoassay and enzymatic procedures on the automatic analyzers, respectively (for blood lipids: AU5800, Beckman Coulter Inc., Japan; for D-dimer: Thrombolyzer XRM, Behnk Elektronik Inc., Germany). According to the instructions of the manufacturer, the normal reference ranges for D-dimer, TG, TC, HDL-C, and LDL-C are 0–1.5 mg/L, 0.51–1.7, 3–5.7, 1.03–1.55, and 1.89–4.21 mmol/L, respectively. Basic information regarding the enrolled mother and their babies, namely, maternal age, height, weight, blood pressure, lifestyle, history of pre-gestational disease, gestational age, gravidity, parity, neonatal sex, birthweight, birth length and pregnancy outcomes were collected from the clinical records. No observational subjects reported smoking, drinking alcohol, and taking illegal drugs during pregnancy.

### Outcome Definition

According to the birthweight, babies were stratified into macrosomia (>4,000 g), normal birthweight (NBW) (2,500–4,000 g) and low birth weight (LBW) (<2,500 g) groups (21). Large for gestational age (LGA) and small for gestational age (SGA) were defined as a live-born baby above the 90th percentile and below the 10th percentile of birthweight for gestational age in a Chinese reference, respectively (22).

### Statistical Methods

The EmpowerStats statistical software version 2.2 ((X&Y Solutions Inc., USA) was used for statistical analysis. Descriptive statistics for demographics and birth outcomes were calculated. Normally and non-normally distributed continuous variables were expressed as the means ± standard deviation (SD) and medians (interquartile range, IQR), and ANOVA, Kruskal–Wallis and Chi-square tests were used to compare the means, medians and proportions of the demographic characteristics in the macrosomia, NBW and LBW mother–newborn pairs. TG, TC, HDL-C, LDL-C, and D-dimer in the LBW, NBW, and macrosomia groups were compared by Kruskal–Wallis test. General linear analysis was used to explore the association between the levels of D-dimer and blood lipids and fetal growth indices (birth length, birthweight, and gestational age). Logistic regression analysis was applied to investigate the associations of macrosomia with traditional risk factors and laboratory tests. To evaluate the performances of D-dimer, blood lipids, traditional risk factors and logistic models in predicting macrosomia, a receiver operator characteristic (ROC) curves analysis was conducted. The best cut-off points or thresholds were determined by calculating and comparing Youden index. Areas under curve (AUC) for risk factors and models were presented to compare the predictive powers. The models were adjusted for the following dichotomous variables: GDM and sex of the infant (male/female), as continuous variables, maternal age, gravidity, parity, gestational age, body mass index (BMI), systolic and diastolic blood pressure (BP) at delivery, were included in the models.

## Results

The population characteristics of pregnant women who delivered NBW babies and those who delivered macrosomia/LBW babies are presented in **Table 1**. A total of 10,396 women with singleton pregnancy were included in our study, 847 of whom complicated with GDM, giving an incidence of 8.1%. Of the 10,396 single gestation live births, the median (interquartile range, IQR) birth weight was 3,360 g (3,080–3,650 g) with a proportion of 3.7% (380) LBW babies and 7.2% (750) macrosomia; 855 (8.2%) were classified as SGA and 1,595 (15.3%) as LGA (**Table S1**). Maternal age, BMI, gravidity, parity, gestational age, delivery mode, the incidence of GDM and PTB, season, and fetal gender were significantly different in the LBW/macrosomia group compared to those in the NBW group. In contrast, maternal blood pressure did not differ among the three groups. When comparing the SGA/LGA and AGA groups, similar differences were observed. Compared to those who delivered NBW babies, women who delivered macrosomia had higher levels of D-dimer and triglyceride and lower TC, HDL-C, and LDL-C levels (median for: D-dimer, 1.27 vs. 1.03 mg/L; triglyceride, 4.03 vs. 3.55 mmol/L; HDL-C, 1.60 vs. 1.72 mmol/L; LDL-C, 3.19 vs. 3.32 mmol/L; TC, 6.20 vs. 6.33 mmol/L; all *P <*0.001; **Table 2**). The association between fetal development and maternal levels of D-dimer and blood lipids are shown in **Table 3**. Adjusted linear regression models showed that a 1-mg/L increase in maternal D-dimer at hospital birth was associated with a 67-g increase in the birthweight (95% CI: 57 to 77) and a 1-mmol/L increase in maternal triglyceride with a 28-g increase in the birthweight (95% CI: 24 to 32). Additionally, there was a significantly negative association between birth weight and HDL-C (−117 g; 95% CI: −138 to −97) for a 1-mmol/L increase. D-dimer was positively associated with gestational age and length at birth, with estimated mean increases of 0.10 week (95% CI: 0.07, 0.13) and 0.06 cm (95% CI: 0.04, 0.08), respectively, for a 1-mg/L increase. Multivariate logistic regression analysis showed that macrosomia shared some of the same factors with LGA babies, namely, maternal gravidity, parity, gestational age at birth, GDM, fetal sex, D-dimer, TG and HDL-C. In the crude regression models, maternal LDL-C and TC levels were negatively associated with macrosomia and LGA. However, these associations lost their significance after adjusting for potential confounders. In addition, maternal BMI increased the risk of macrosomia after correcting for potential confounders (adjusted OR: 1.52, 95% CI: 1.02–2.26), but were not associated with the risk of LGA (**Table 4**).

**Table 1:** Maternal and neonatal characteristics in the study populations according to the birthweight.

	LBW (N = 380)	NBW (N = 9,266)	Macrosomia (N = 750)	*P-*value
Maternal characteristics				
Maternal age at delivery (years)	27.6 ± 4.5	28.5 ± 4.3	29.1 ± 4.5	<0.001
<25	99 (26.1%)	1,334 (14.4%)	100 (13.3%)	<0.001
25–34	251 (66.1%)	6,928 (74.8%)	545 (72.7%)	
≥35	30 (7.9%)	1,004 (10.8%)	105 (14.0%)	
BMI at delivery (kg/m^2^)	26.0 ± 3.4	27.1 ± 3.2	29.4 ± 3.3	<0.001
<25	155 (43.8%)	2,428 (26.4%)	51 (6.8%)	<0.001
25–29.9	120 (33.9%)	3,522 (38.3%)	214 (28.6%)	
≥30	79 (22.3%)	3,242 (35.3%)	482 (64.5%)	
Gravidity				
<3	261 (68.7%)	6,646 (71.7%)	494 (65.9%)	0.002
≥3	119 (31.3%)	2,620 (28.3%)	256 (34.1%)	
Parity				
No child	229 (60.3%)	5,610 (60.5%)	397 (52.9%)	<0.001
≥1 child	151 (39.7%)	3,656 (39.5%)	353 (47.1%)	
Gestational age at delivery (week)	34.0 ± 2.9	38.9 ± 1.2	39.5 ± 1.0	<0.001
Systolic BP at delivery (mmHg)	120 (110–130)	120 (110–128)	120 (110–128)	0.166
Diastolic BP at delivery (mmHg)	74 (70–80)	72 (70–78)	72 (70–79)	0.130
Delivery mode				
Vaginal delivery	230 (60.5%)	5,525 (59.6%)	304 (40.5%)	<0.001
Cesarean section	150 (39.5%)	3,741 (40.4%)	446 (59.5%)	
PTB	289 (76.1%)	338 (3.6%)	1 (0.1%)	<0.001
GDM	25 (6.6%)	710 (7.7%)	112 (14.9%)	<0.001
Season				
Spring	66 (17.4%)	1,963 (21.2%)	183 (24.4%)	0.007
Summer	97 (25.5%)	2,388 (25.8%)	179 (23.9%)	
Autumn	113 (29.7%)	2,611 (28.2%)	175 (23.3%)	
Winter	104 (27.4%)	2,304 (24.9%)	213 (28.4%)	
Newborn characteristics				
Sex				
Female	169 (44.5%)	4,476 (48.3%)	261 (34.8%)	<0.001
Male	211 (55.5%)	4,790 (51.7%)	489 (65.2%)	
Birth length (cm)	45.2 ± 3.5	50.0 ± 0.4	51.1 ± 1.2	<0.001
Birthweight (g)	2,200 (1,845–2,363)	3,350 (3,100–3,590)	4.180 (4.080–4.350)	<0.001
SGA	152 (40.0%)	703 (7.6%)	0 (0.0%)	<0.001
AGA	208 (54.7%)	7,682 (82.9%)	56 (7.5%)	
LGA	20 (5.3%)	881 (9.5%)	694 (92.5%)	

LBW, low birthweight; NBW, normal birthweight; BMI, body mass index; BP, blood pressure; PTB, pre-term birth; GDM, gestational diabetes mellitus; SGA/AGA/LGA, small/appropriate/large for gestational age; IQR, interquartile range.

Data are presented as median (IQR), mean ± SD and N (%).

**Table 2:** Maternal D-dimer and blood lipid levels before childbirth according to the birthweight.

	LBW	NBW	Macrosomia	P-value	SGA	AGA	LGA	P-value
D-dimer (mg/L)	0.72 (0.47–1.12)	1.03 (0.66–1.55)	1.27 (0.84–1.92)	<0.001	0.84 (0.57–1.32)	1.03 (0.65–1.54)	1.19 (0.77–1.80)	<0.001
Triglyceride (mmol/L)	2.88 (2.22–3.92)	3.55 (2.77–4.58)	4.03 (3.22–5.29)	<0.001	3.21 (2.48–4.27)	3.52 (2.75–4.55)	3.87 (3.08–5.4)	<0.001
HDL-C (mmol/L)	1.71 (1.46–1.93)	1.72 (1.50–1.96)	1.60 (1.41–1.82)	<0.001	1.79 (1.56–2.05)	1.72 (1.50–1.96)	1.63 (1.42–1.86)	<0.001
LDL-C (mmol/L)	3.14 (2.58–3.76)	3.32 (2.75–3.95)	3.19 (2.59–3.80)	<0.001	3.35 (2.81–4.01)	3.32 (2.76–3.94)	3.20 (2.60–3.88)	<0.001
Total cholesterol (mmol/L)	5.96 (5.26–6.72)	6.33 (5.59–7.13)	6.20 (5.41–6.96)	<0.001	6.29 (5.62–7.19)	6.32 (5.58–7.11)	6.20 (5.47–7.06)	0.002

Data was presented as median (IQR) and N(%).

IQR, interquartile range; LBW, low birthweight; NBW, normal birthweight; SGA/AGA/LGA, small/appropriate/large for gestational age; HDL-C, high-density lipoprotein cholesterol; LDL-C, low-density lipoprotein cholesterol.

**Table 3:** Regression coefficients [β (95% CI)] for neonatal development associated with maternal D-dimer and blood lipid levels.

Variables	Gestational age (weeks)^a^	Birth length (cm)^b^	Birth weight (g)^b^
Unadjusted			
D-dimer (mg/L)	0.11 (0.08, 0.15)**	0.11 (0.08, 0.13)**	67.12 (57.20, 77.05)**
Triglyceride (mmol/L)	0.07 (0.05, 0.08)**	0.07 (0.06, 0.09)**	46.66 (41.59, 51.72)**
HDL-C (mmol/L)	0.08 (−0.01, 0.17)	−0.04 (−0.11, 0.03)	−143.48 (−169.78, −117.18)**
LDL-C (mmol/L)	0.06 (0.03, 0.10)**	0.02 (0.00, 0.05)	−19.64 (−29.70, −9.57)**
Total cholesterol (mmol/L)	0.07 (0.04, 0.09)**	0.05 (0.03, 0.07)**	−0.50 (−8.20, 7.20)
Adjusted			
D-dimer (mg/L)	0.10 (0.07, 0.13)**	0.06 (0.04, 0.08)**	56.47 (48.70, 64.24)**
Triglyceride (mmol/L)	0.07 (0.05, 0.08)**	0.03 (0.02, 0.04)**	28.04 (23.99, 32.09)**
HDL-C (mmol/L)	0.04 (−0.05, 0.13)	−0.05 (−0.11, 0.00)	−117.16 (−137.79, −96.52)**
LDL-C (mmol/L)	0.05 (0.01, 0.08)**	0.03 (0.01, 0.05)*	4.44 (−3.56, 12.45)
Total cholesterol (mmol/L)	0.06 (0.03, 0.09)**	0.03 (0.02, 0.05)**	9.23 (3.12, 15.34)**

HDL-C, high-density lipoprotein cholesterol; LDL-C, low-density lipoprotein cholesterol; BMI, body mass index; BP, blood pressure; GDM, gestational diabetes mellitus.

a Adjusted for maternal age, gravidity, parity, BMI and BP at delivery, GDM and fetal sex.

b Additionally corrected for gestational age.

*P <0.05, **P <0.01.

**Table 4:** The risk factors associated with the incident macrosomia and LGA neonates in late pregnancy.

Variables	Macrosomia^a^				LGA^b^			
	Univariate		Multivariate		Univariate		Multivariate	
	OR (95% CI)	*P*-value	OR (95% CI)	*P-*value	OR (95% CI)	*P*-value	OR (95% CI)	*P-*value
Maternal age (years)	1.03 (1.01, 1.05)	<0.001	1.02 (1.00, 1.04)	0.093	1.08 (1.06, 1.09)	<0.001	1.04 (1.02, 1.05)	<0.001
Height (cm)	1.08 (1.06, 1.10)	<0.001	1.20 (1.04, 1.38)	0.013	1.05 (1.04, 1.06)	<0.001	1.05 (0.95, 1.17)	0.327
Weight (kg)	1.08 (1.07, 1.09)	<0.001	0.92 (0.79, 1.07)	0.282	1.07 (1.06, 1.07)	<0.001	1.03 (0.92, 1.16)	0.596
BMI (kg/m^2^)	1.22 (1.19, 1.24)	<0.001	1.52 (1.02, 2.26)	0.038	1.18 (1.17, 1.20)	<0.001	1.10 (0.81, 1.49)	0.533
Gravidity	1.13 (1.07, 1.20)	<0.001	1.10 (1.01, 1.20)	0.036	1.26 (1.21, 1.32)	<0.001	1.07 (1.01, 1.14)	0.030
Parity	1.30 (1.14, 1.47)	<0.001	1.27 (1.05, 1.54)	0.014	1.74 (1.59, 1.91)	<0.001	1.31 (1.15, 1.51)	<0.001
Gestational age at delivery (week)	1.56 (1.46, 1.68)	<0.001	1.75 (1.62, 1.89)	<0.001	0.87 (0.84, 0.90)	<0.001	0.84 (0.81, 0.87)	<0.001
GDM	2.12 (1.71, 2.62)	<0.001	1.90 (1.49, 2.42)	<0.001	2.29 (1.95, 2.69)	<0.001	1.67 (1.40, 2.00)	<0.001
Neonatal sex (male)	1.75 (1.50, 2.05)	<0.001	2.06 (1.74, 2.43)	<0.001	1.64 (1.47, 1.84)	<0.001	1.78 (1.58, 2.01)	<0.001
D-dimer (mg/L)	1.24 (1.16, 1.33)	<0.001	1.33 (1.23, 1.43)	<0.001	1.23 (1.17, 1.30)	<0.001	1.35 (1.27, 1.43)	<0.001
Triglyceride (mmol/L)	1.16 (1.12, 1.19)	<0.001	1.14 (1.05, 1.23)	0.002	1.13 (1.11, 1.17)	<0.001	1.12 (1.05, 1.18)	<0.001
HDL-C (mmol/L)	0.31 (0.25, 0.39)	<0.001	0.35 (0.24, 0.51)	<0.001	0.43 (0.36, 0.51)	0.004	0.47 (0.36, 0.62)	<0.001
LDL–C (mmol/L)	0.81 (0.74, 0.88)	<0.001	1.16 (0.91, 1.48)	0.231	0.85 (0.80, 0.91)	<0.001	1.17 (0.97, 1.40)	0.102
Total cholesterol (mmol/L)	0.90 (0.84, 0.96)	0.001	0.99 (0.79, 1.23)	0.902	0.93 (0.89, 0.98)	0.003	0.98 (0.83, 1.16)	0.811

OR, odds ratio; CI, confidence interval; BMI, body mass index; GDM, gestational diabetes mellitus; HDL-C, high-density lipoprotein cholesterol; LDL-C, low-density lipoprotein cholesterol. ^a^Excluding 380 neonates with low birthweight (<2,500 g). ^b^Excluding 853 SGA neonates.

**Figure 1:**
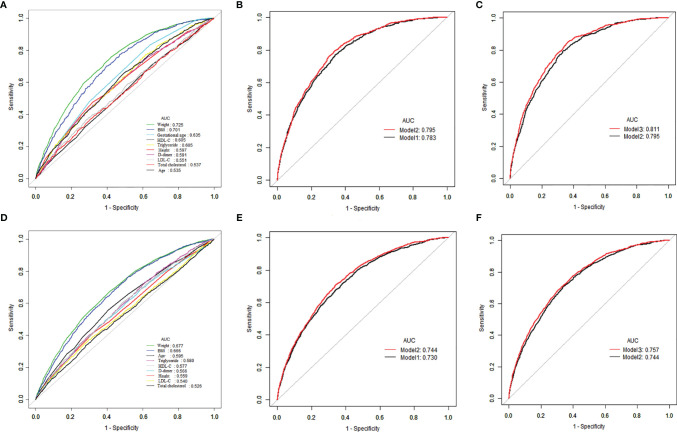
ROC curves to compare the effects of different variables in predicting macrosomia and LGA infants in late pregnancy. Model 1, conventional model, namely, maternal age, BMI, gravidity, parity, gestational age, systolic and diastolic BP at hospital admission and fetal sex; Model 2, model 1 plus D-dimer; Model 3, model 2 plus blood lipids. **(A)** The prediction of macrosomia by individual variables. **(B, C)** Predictive ability of different models of macrosomia. **(D)** The prediction of LGA by individual variables. **(E, F)** Predictive ability of different models of LGA.

An ROC curve analysis was performed to examine the predictive ability of D-dimer, blood lipids levels at hospital birth and traditional risk factors (**Figure 1**). Among the traditional risk factors, the AUC of maternal weight at hospital birth was 0.725 and was greater than those for others (**Table 5**). Among the laboratory test items, the AUC for HDL-C and TG were the same at 0.605 and were significantly higher than those for LDL-C and TC (all *P <*0.001). Although the AUC of D-dime (0.591) was lower than those for HDL-C and TG; the difference did not remain significant (*P =* 0.344). The optimal cut-off point of HDL-C for predicting macrosomia was 1.72 mmol/L, with a sensitivity of 65.47%, specificity of 50.84%, positive predictive value (PPV) of 9.73% and negative predictive value (NPV) of 94.79%. In addition, prediction models for macrosomia combining clinical risk factors and biochemical indicators were further determined. In model 1, the traditional risk factors included maternal age, height, weight, gestational age, gravidity, parity, blood pressure, GDM, and fetal sex. The AUC for the model 1 was 0.783 (95% CI 0.767, 0.799). Incorporating D-dimer and blood lipids into the model 1 significantly increased the AUC to 0.795 (95% CI 0.780, 0.810) and 0.811 (95% CI 0.796, 0.826), respectively. To predict LGA using these models, similar results were found (**Table 6**).

**Table 5:** Accuracy of different models and variables in late pregnancy to predict macrosomia.

Variables	AUC	95% CI	*P*-value	Best threshold	Sensitivity (%)	Specificity (%)	PPV (%)	NPV (%)
Weight (kg)	0.725	0.708–0.743	<0.001	71.75	75.77	57.82	12.73	96.71
BMI (kg/m^2^)	0.701	0.683–0.719	<0.001	26.96	77.38	52.95	11.79	96.64
Gestational age (week)	0.635	0.617–0.653	0.039	38.5	83.33	35.83	9.51	96.37
HDL-C (mmol/L)	0.605	0.585–0.626		1.72	65.47	50.84	9.73	94.79
Triglyceride (mmol/L)	0.605	0.584–0.625	0.968	3.18	77.07	38.52	9.21	95.40
Height (cm)	0.597	0.576–0.618	0.593	163.5	47.60	68.08	10.78	94.13
D-dimer (mg/L)	0.591	0.570–0.612	0.344	1.18	55.20	58.74	9.78	94.18
LDL-C (mmol/L)	0.551	0.529–0.573	<0.001	2.84	36.41	71.89	9.49	93.32
Total cholesterol (mmol/L)	0.537	0.516–0.559	<0.001	5.75	36.53	69.85	8.93	93.15
Age (years)	0.535	0.514–0.556	<0.001	26.5	70.53	35.27	8.10	93.67
Model 1	0.783	0.767–0.799	<0.001	−2.66	76.97	66.80	15.54	97.24
Model 2	0.794	0.780–0.810		−2.70	85.03	58.68	16.15	97.57
Model 3	0.811	0.796–0.826	<0.001	−2.81	84.54	63.96	16.03	98.07

AUC, area under the curve; CI, confidence interval; PPV, positive predictive value; NPV, negative predictive value; BMI, body mass index; FDP, fibrin/fibrinogen degradation products; HDL-C, high density lipoprotein cholesterol; LDL-C, low density lipoprotein cholesterol; TG, triglyceride; TC, total cholesterol. P-values express the significance of differences between HDL-C and 9 other variables or the difference between Model 2 and Model 1/3.

**Table 6:** Accuracy of different models and variables in late pregnancy to predict LGA.

Variables	AUC	95% CI	*P*-value	Best threshold	Sensitivity (%)	Specificity (%)	PPV (%)	NPV (%)
Weight (kg)	0.677	0.663–0.691	<0.001	71.75	67.81	58.20	24.57	90.00
BMI (kg/m^2^)	0.666	0.651–0.680	<0.001	26.96	76.09	48.53	22.90	90.99
Age (years)	0.595	0.579–0.610	0.156	28.5	55.74	59.38	21.59	86.98
Triglyceride (mmol/L)	0.580	0.565–0.595		3.17	73.17	38.81	19.36	87.81
HDL-C (mmol/L)	0.577	0.562–0.592	0.786	1.71	59.81	51.90	19.97	86.55
D-dimer (mg/L)	0.566	0.551–0.582	0.210	1.20	49.91	60.41	20.21	85.72
Height (cm)	0.559	0.544–0.575	0.068	163.5	41.69	67.69	20.59	85.24
LDL-C (mmol/L)	0.540	0.524–0.556	<0.001	2.88	37.34	70.13	20.09	84.77
Total cholesterol (mmol/L)	0.526	0.511–0.542	<0.001	5.84	38.93	66.74	19.03	84.48
Model 1	0.730	0.717–0.743	<0.001	−1.66	67.99	65.69	28.50	91.07
Model 2	0.744	0.731–0.756		−1.69	66.10	70.77	29.61	91.82
Model 3	0.757	0.744–0.769	<0.001	−1.60	67.72	70.16	31.38	91.52

AUC, area under the curve; CI, confidence interval; PPV, positive predictive value; NPV, negative predictive value; BMI, body mass index; FDP, fibrin/fibrinogen degradation products; HDL-C, high density lipoprotein cholesterol; LDL-C, low density lipoprotein cholesterol; TG, triglyceride; TC, total cholesterol. P-values express the significance of differences between triglyceride and 8 other variables or the difference between Model 2 and Model 1/3.

## Discussion

This population‐based cohort study comprehensively displayed maternal D-dimer and lipid profiles before childbirth in GDM and normal pregnancies and explored the associations of macrosomia/LGA births with maternal D-dimer and lipid concentrations, and developed clinical models for antenatal prediction of the birth of macrosomia/LGA. The main findings of this study were that both D-dimer and lipid levels (TG and HDL-C) had independent and significant effects on the risk of delivering macrosomia/LGA neonates. In addition, D-dimer, lipid levels, and maternal characteristics, namely, age, height, weight, and BMI, were significant predictors of macrosomia/LGA. More importantly, incorporating D-dimer and lipid levels into the prediction model including maternal clinical information could gradually improve the predictive capacity for the birth of macrosomia/LGA in GDM and normal pregnancies.

Fetal macrosomia is associated with adverse perinatal outcomes, which complicates about 5–20% of all pregnancies in developed countries (2). With the rapid growth of the Chinese economy in the past four decades, the rate of fetal macrosomia has increased accordingly. For example, one study on secular trends of fetal macrosomia in southeast China demonstrated an increase from 6.0% in 1994 to 7.8% in 2005 (23). Another study conducted in Harbin, a northern city of China, reported that the incidence of macrosomia had increased from 8.3% in 2001 to 10.5% in 2005 (24). However, due to the changes in dietary structure, healthcare and sanitation in the recent decade, the prevalence of macrosomia has shown a downward trend since 2010. For example, a hospital-based survey conducted in 14 provinces in China, covering a wide range of geographic areas, demonstrated that the total prevalence of macrosomia in 2011 was 7.3% (25). The incidence of macrosomia in Beijing, China was 8.0% in 2007–2011 and reduced to 6.8% in 2011–2013 (26). The same incidence of fetal macrosomia was observed in Shaanxi province of Northwest China in 2010–2013 (27). In rural areas of Henan province of central China, the rate of fetal macrosomia decreased by 31.3% from 8.0% in 2013 to 5.5% in 2017 (28). In the present study, the prevalence of macrosomia in GDM and uncomplicated pregnancies was 7.2%, and was higher than recent reports. The discrepancy could be explained by study location and composition of the study population. A questionnaire survey conducted among women in Xi ‘an, a central city of China, demonstrated that the prevalence of fetal macrosomia was lower in rural–urban areas than in the central district (29). According to the practice bulletin of the American College of Obstetricians and Gynecologists on fetal macrosomia, multipara is an important risk factor (5). More than 40% of the participants in this study were multiparous and was higher than those in previous studies from China because of the implementation of the Two-Child Policy since 2016. The present study noted that multiparous women had a significantly higher rate of macrosomia compared to nulliparous women (8.5% vs. 6.4%). In order to reduce the occurrence of fetal macrosomia in multiparas women, pre-pregnancy education and pregnancy guidance should be greatly strengthened. In addition, the findings of prevalence of fetal macrosomia might differ because this study excluded a number of pregnancies complicated by PE (379 cases). Our previous reports suggested that women with PE had a lower incidence of fetal macrosomia compared to those without pregnancy complications among individuals who underwent serum screening for Down syndrome (3.9% vs. 7.8%) or noninvasive prenatal examination (5.8% vs. 6.9%) (30, 31).

Fetal growth depends on a complex interaction of various environmental and genetic factors. Consequently, it is difficult to predict pregnancy at risk of overgrowth. Identification of risk factors for fetal macrosomia is an increasingly relevant issue. These influences can be divided into changeable and unchangeable factors in clinical practice (32). Consistent with a previous review by Jennifer et al. in 2012, unmodifiable factors, such as maternal height, gravidity, parity, and fetal gender, and modifiable factors, namely, maternal BMI and gestational age before delivery, and GDM were proved to be independent risk factors for macrosomia in this study (33). In addition, our findings emphasized maternal D-dimer and TG levels before delivery are positively correlated with fetal birthweight, and are positively associated with the risk of fetal macrosomia and LGA, which are consistent with previous studies conducted in China (19, 34). Furthermore, our results suggested that the higher HDL-C levels decreased the birthweight and are inversely associated with the risk of fetal macrosomia and LGA, in accordance with the findings of some publications (19, 34).

Prenatal prediction of fetal macrosomia is crucial for clinicians to determine delivery mode. It is even more important in the case of pregnancies complicated with maternal diabetes, since the rate of macrosomia is as high as 2–3 times, with higher incidence of shoulder dystocia (30, 31, 35). Previous studies on screening pregnant women for fetal macrosomia reported two types of practical methods with overall low prediction rate, namely, ultrasonography and maternal physical examination (maternal abdomen and basal height) (5). When comparing the efficiency of these methods, it is concluded that none of the methods show obvious advantages over others (5). Importantly, these available methods have their common limitation of imprecision, showing that the sensitivity and specificity of fetal macrosomal identification are about 55 and 90%, respectively (36). In this study, we developed three models to predict macrosomia delivery by using several significant biomarkers and maternal parameters, without sonographic examination. The sensitivity (76–85%) and specificity (58–66%) of these models have not been significantly improved; however, our predictive models had high NPV values (97–98%), which might be used to exclude fetal macrosomia, and could be beneficial in different situations with limited clinical resources.

The strengths of this prediction study for fetal macrosomia are, first, the examination of a large population of women involved in routine evaluation of D-dimer and lipid levels at hospital admission allowed us to extend the results to substantially larger areas in China; second, the uniform diagnostic criteria for pregnancy outcomes and the same laboratory tests for those biochemical markers reduced possible bias; third, there was clear comparison of the prediction ability of clinical characteristics, D-dimer and lipid profiles; and, fourth, the presentation of different prediction models for antenatal prediction of macrosomia/LGA neonates. The limitations of the present study should be mentioned. First of all, our data did not record information regarding previous history of GDM, macrosomia, pre-pregnancy weight, and gestational weight gain (GWG), and dietary intake. These impact factors increase the risk of macrosomia/LGA (37, 38). The prediction models could have been more effective if these factors were included. Secondly, in this study, the best predictive model with an AUC of 0.811 was commonly regarded as fairly good. The model might be used to rule out the risk of macrosomia birth in GDM and healthy pregnancies due to its high NPV (98.07%); however, the false positive results caused by low PPV (13.36%) should have been noted when determining the women who will give birth of macrosomia. An inaccurate identification might contribute to unnecessary interventions, such as an increase in the proportion of cesarean section. Therefore, how to increase the PPV of prediction model without reducing NPV is worthy of further study.

In conclusion, this study comprehensively displayed maternal D-dimer level and lipid profile at hospital admission for delivery and suggested that D-dimer and lipid levels could be independent and significant predictors of macrosomia/LGA. In addition, the present study demonstrated that the combination of D-dimer and lipid levels with conventional risk factors might improve the prediction performance of macrosomia/LGA in GDM and normal pregnancies.

## Data Availability Statement

The raw data supporting the conclusions of this article will be made available by the authors, without undue reservation.

## Ethics Statement

The studies involving human participants were reviewed and approved by the Changzhou Maternity and Child Health Care Hospital Ethics Committee. The patients/participants provided their written informed consent to participate in this study.

## Author Contributions

BY and JZ conceived and designed this study. XY wrote the manuscript. XH and CJ collected the data. HW interpreted revised the reports. All authors listed have made a substantial, direct, and intellectual contribution to the work and approved it for publication.

## Funding

This work was supported by the Changzhou Science and Technology Support Project (Social Development: CE20205028), and the Jiangsu Maternal and Child Health Research Projects (F201842).

## Conflict of Interest

The authors declare that the research was conducted in the absence of any commercial or financial relationships that could be construed as a potential conflict of interest.

## Publisher’s Note

All claims expressed in this article are solely those of the authors and do not necessarily represent those of their affiliated organizations, or those of the publisher, the editors and the reviewers. Any product that may be evaluated in this article, or claim that may be made by its manufacturer, is not guaranteed or endorsed by the publisher.

## Supplementary Material

Supplementary Table 1 Maternal and neonatal characteristics in the study populations according to birthweight. SGA/AGA/LGA, small/appropriate/large for gestational age; BMI, body mass index; BP, blood pressure; PTB, pre-term birth; GDM, gestational diabetes mellitus; LBW, low birthweight; NBW, normal birthweight; IQR, interquartile range. Data are presented as median (IQR), mean ± SD and N(%).

## Abbreviations

LBW, low birth weight; LGA, large for gestational age; SGA, small for gestational age; PE, pre-eclampsia; PTB, preterm birth; GDM, gestational diabetes mellitus; ICP, intrahepatic cholestasis of pregnancy; PIH, pregnancy induced hypertension; NPC, non-pregnancy complication; VTE, venous thromboembolism; BMI, body mass index; OR, odds ratio; CI, confidence interval.
