# Supplementary material for: Investigation and Application of Risk Factors of Macrosomia Based on 10,396 Chinese Pregnant Women
Source: Front Endocrinol (Lausanne). 2022 Apr 26;13:837816. doi: 10.3389/fendo.2022.837816 (PMC9088515; doi:10.3389/fendo.2022.837816)
Supplement: Supplementary Table 1 — Maternal and neonatal characteristics in the study populations according to birthweight. SGA/AGA/LGA, small/appropriate/large for gestational age; BMI, body mass index; BP, blood pressure; PTB, pre-term birth; GDM, gestational diabetes mellitus; LBW, low birthweight; NBW, normal birthweight; IQR, interquartile range. Data are presented as median (IQR), mean ± SD and N(%). [file Table_1.pdf]

Table S1 Maternal and neonatal characteristics in the study populations according to birthweight.

	SGA (N= 855)	AGA (N= 7946)	LGA (N= 1595)	*P* value
Maternal characteristics				
Maternal age at delivery (years)	27.6 ± 4.2	28.4 ± 4.3	29.9 ± 4.56	<0.001
<25	170 (19.88%)	1196 (15.05%)	167 (10.47%)	
25–34	621 (72.63%)	5957 (74.97%)	1146 (71.85%)	<0.001
≥35	64 (7.49%)	793 (9.98%)	282 (17.68%)	
BMI at delivery (kg/m2)	25.5 ± 3.1	27.1 ± 3.1	28.9 ± 3.3	<0.001
<25	401 (47.51%)	2073 (26.35%)	160 (10.12%)	
25–29.9	286 (33.89%)	3066 (38.97%)	504 (31.88%)	<0.001
≥30	157 (18.60%)	2729 (34.68%)	917 (58.00%)	
Gravidity				
< 3	674 (78.83%)	5764 (72.54%)	963 (60.38%)	<0.001
≥3	181 (21.17%)	2182 (27.46%)	632 (39.62%)	
Parity				
No child	601 (70.29%)	4904 (61.72%)	731 (45.83%)	<0.001
≥ 1 child	254 (29.71%)	3042 (38.28%)	864 (54.17%)	
Gestational age at delivery (week)	38.9 ± 1.9	38.8 ± 1.57	38.4 ± 1.6	<0.001
Systolic BP at delivery (mmHg)	120 (110–128)	120 (110–128)	120 (110–128)	0.742
Diastolic BP at delivery (mmHg)	73 (70–80)	72 (70–78)	71 (70–79)	0.012
Delivery mode				

Vaginal delivery	603 (70.53%)	4806 (60.48%)	650 (40.75%)	<0.001
Cesarean section	252 (29.47%)	3140 (39.52%)	945 (59.25%)	
PTB	61 (7.13%)	455 (5.73%)	112 (7.02%)	0.053
GDM	37 (4.33%)	570 (7.17%)	240 (15.05%)	<0.001
Season				
Spring	171 (20.00%)	1699 (21.38%)	342 (21.44%)	0.001
Summer	237 (27.72%)	2026 (25.50%)	401 (25.14%)	
Autumn	243 (28.42%)	2268 (28.54%)	388 (24.33%)	
Winter	204 (23.86%)	1953 (24.58%)	464 (29.09%)	
Newborn characteristics				
Sex				
Female	528 (61.75%)	3806 (47.90%)	572 (35.86%)	<0.001
Male	327 (38.25%)	4140 (52.10%)	1023 (64.14%)	
Birth length (cm)	49.1 ± 2.31	49.8 ± 1.0	50.4 ± 1.3	<0.001
Birth weight (g)	2760 (2560–2900)	3330 (3110–3550)	3950 (3730–4160)	<0.001
<2500	152 (40.0%)	703 (7.6%)	0 (0.0%)	<0.001
2500–4000	208 (54.7%)	7682 (82.9%)	56 (7.5%)	
>4000	20 (5.3%)	881 (9.5%)	694 (92.5%)	

Abbreviations: SGA/AGA/LGA small/appropriate/large for gestational age; BMI, body mass index; BP, blood pressure; PTB, pre-term birth; GDM, gestational diabetes mellitus; LBW, low birthweight; NBW, normal birthweight; IQR, interquartile range.

Data are presented as median (IQR), mean ± SD and N(%).
